# Pellagra, the Pellagrous Intestine and Pericolic Veins

**Published:** 1917-12

**Authors:** John L. Jelks

**Affiliations:** Memphis, Tenn.


### PELLAGRA, THE PELLAGROUS INTESTINE AND PERICOLIC VEILS.

**By John L. Jelks, M.D., Memphis, Tenn.**

Dr. John L. Jelks describes Recto-Colonic Conditions in Pellagra, gives his observations in Pellagra, which he has contended for seven years is an infection, which begins in the intestine in a similar manner as does typhoid fever, and contends that in all active or acute cases of Pellagra he finds pathology in the intestines commensurate with other symptoms.

The speaker describes the prodromal and the late symptoms and both the early and late pathology observed by him. The speaker describes the entra-abdominal as well also the gut pathology in the different stages.

The speaker says Pellagra seldom develops in sanitary and screened homes, and believes that sanitation, isolation and the elimination of raw vegetables as a diet and effectual screening will control Pellagra. He refers to the fact that the

4—MEDICAL shrdlu etaoin shrdlu uu negroes and poor whites in this section have more money, and more to eat and a greater variety of diet than they ever had, yet that Pellagra is increasing rapidly in the out-lying districts about Memphis, though it seldom extends over into the sewered and screened section of the city proper. The speaker referred to a recent ordinance that had passed in the City of Memphis requiring doctors to report all cases of Pellagra and to exercise the same precautionary measures in these as in cases of typhoid fever.

